# Long noncoding RNA LINC00461 induced osteoarthritis progression by inhibiting miR-30a-5p

**DOI:** 10.18632/aging.102839

**Published:** 2020-03-10

**Authors:** Yuanmin Zhang, Longfei Ma, Chengqun Wang, Lina Wang, Yanxia Guo, Guodong Wang

**Affiliations:** 1 Department of Orthopedics, Affiliated Hospital of Jining Medical University, Jining 272029, Shandong, China; 2 Department of Orthopedics, The Second Hospital of Shandong University, Jinan 250000, Shandong, China

**Keywords:** osteoarthritis, LINC00461, miR-30a-5p, chondrocytes

## Abstract

Mounting studies have shown that long noncoding RNAs (lncRNAs) play important roles in the development and occurrence of several human diseases. However, the role of LINC00461 in osteoarthritis (OA) remains obscure. A CCK-8 assay was performed to detect cell viability, and qRT-PCR analysis was used to measure mRNA expression. The targeting by miR-30a-5p of the LINC00461 3’UTR was detected using a luciferase reporter assay. Our data indicated that the inflammatory mediators IL-6 and TNF-α induced LINC00461 expression in chondrocytes and that the expression of LINC00461 was upregulated in OA tissues. Furthermore, we showed that TNF-α and IL-6 suppressed the expression of miR-30a-5p and that miR-30a-5p expression was lower in OA tissues than in normal samples. The expression level of miR-30a-5p in OA tissues was negatively related to LINC00461 expression. In addition, we showed that LINC00461 directly interacted with miR-30a-5p in chondrocytes. Elevated expression of LINC00461 induced chondrocyte proliferation, cell cycle progression, inflammation, and extracellular matrix (ECM) degradation. However, we demonstrated that ectopic expression of miR-30a-5p suppressed cell growth, cell cycle progression, inflammation and ECM degradation. Finally, we found that overexpression of LINC00461 enhanced chondrocyte proliferation, cell cycle progression, inflammation, and ECM degradation by downregulating miR-30a-5p. These data demonstrated that LINC00461 may modulate the development of OA by suppressing miR-30a-5p expression in chondrocytes. We propose that LINC00461 and miR-30a-5p may be potential therapeutic and diagnostic targets for OA.

## INTRODUCTION

Osteoarthritis (OA) is characterized by inflammatory and degenerative processes that affect articular cartilage and joints and is the 4^th^ leading cause of disability and pain worldwide [1–4]. Approximately 18% of females and 10% of males over sixty years old are diagnosed with OA [5–7]. The pathology and mechanism of OA could be regulated through the processing of both environmental and genetic information [8–11]. Chondrocytes are activated via growth factors and cytokines to induce abnormal differentiation and catabolism, which results in extracellular matrix (ECM) degradation [6, 12–16]. For example, IL-6 was found to be involved in the development of OA [17, 18]. Thus, it is imperative to explore the molecular mechanism of OA.

Long noncoding RNAs (lncRNAs) are novel noncoding RNAs (ncRNAs) that are longer than 200 nucleotides in length with no or limited protein-coding potential [19–22]. A growing number of studies have revealed that lncRNAs are deregulated in several diseases, such as tumors, intervertebral disc degeneration, infection and OA [23–27]. LncRNAs have been shown to participate in a number of cell biological processes, including apoptosis, stem cell differentiation, proliferation, ECM degradation and invasion [28–31]. Recently, the novel lncRNA LINC00461 was found to play important roles in the progression of several tumors, including glioma, multiple myeloma and breast cancer [32–34]. For instance, Yang et al. [32]. showed that LINC00461 was overexpressed in glioma samples and that knockdown of LINC00461 inhibited the expression of cyclin D1/A/E and cell growth in glioma cells partly by regulating the PI3K/AKT and MAPK/ERK signaling pathways. In addition, LINC00461 knockdown suppressed miR-9 expression and the related genes TMEM161B and MEF2C. However, the functional role of LINC00461 in OA development remains unknown.

In our research, we aimed to study the role of LINC00461 in OA development. We first confirmed that the inflammatory mediators IL-6 and TNF-α induced LINC00461 expression in chondrocytes and that the expression of LINC00461 was upregulated in OA tissues. Elevated expression of LINC00461 induced chondrocyte proliferation, cell cycle progression, inflammation, and extracellular matrix (ECM) degradation.

## RESULTS

### TNF-α and IL-6 induced the expression of LINC00461 in chondrocytes

**Figure 1:**
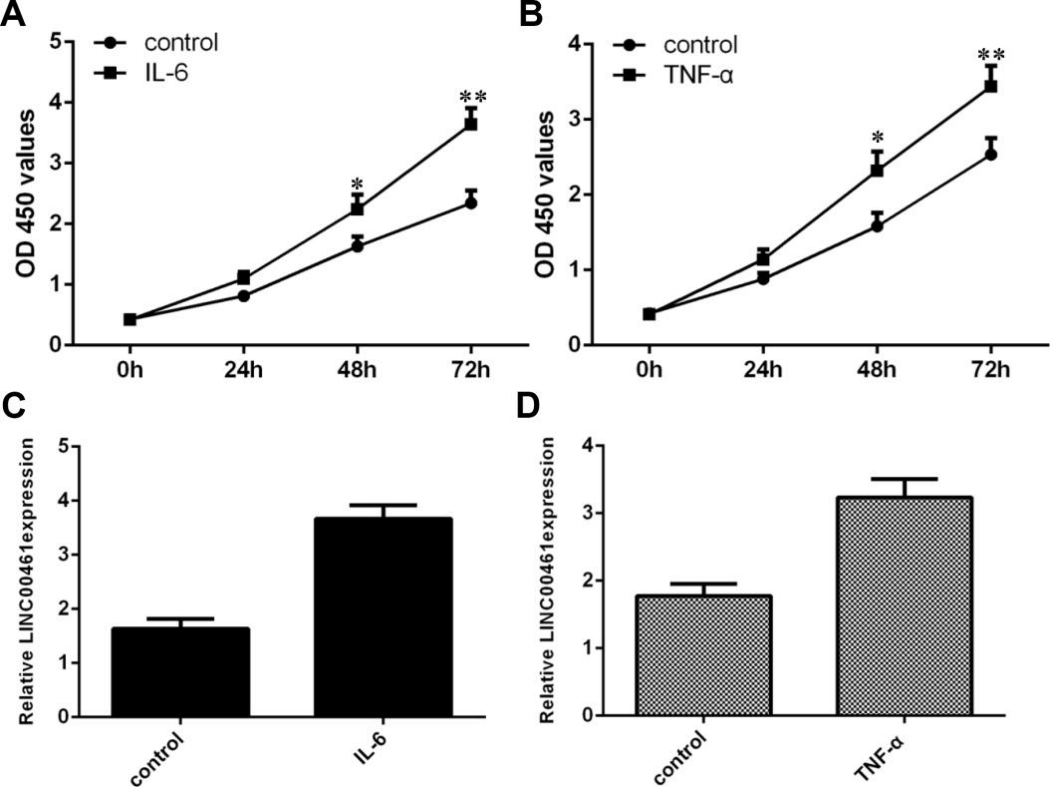
**TNF-α and IL-6 induced the expression of LINC00461 in chondrocytes.** (**A**) Cell proliferation was determined by CCK-8 analysis. (**B**) TNF-α promoted chondrocyte growth, as demonstrated by CCK-8 analysis. (**C**) IL-6 enhanced the expression of LINC00461 in chondrocytes, as shown by qRT-PCR. GAPDH was used as the internal control. (**D**) TNF-α induced LINC00461 expression in chondrocytes. *p<0.05 and **p<0.01.

To explore the effect of TNF-α and IL-6 on LINC00461 expression in OA, we treated chondrocytes with TNF-α and IL-6. We first confirmed that IL-6 could significantly induce chondrocyte proliferation by using a CCK-8 assay (Figure 1A). We also found that TNF-α significantly promoted chondrocyte growth using CCK-8 analysis (Figure 1B). In addition, we indicated that IL-6 enhanced the expression of LINC00461 in chondrocytes (Figure 1C). We demonstrated that TNF-α induced LINC00461 expression in chondrocytes (Figure 1D).

### The expression of LINC00461 was upregulated in OA tissues

**Figure 2:**
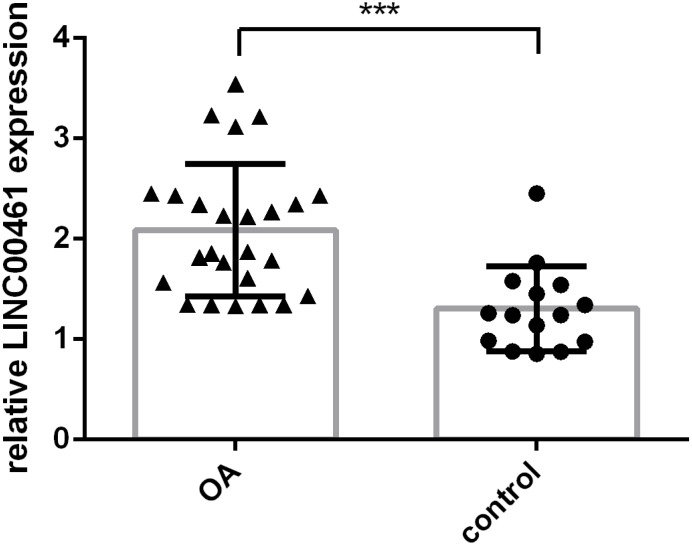
**The expression of LINC00461 was upregulated in OA tissues.** LINC00461 expression was detected in OA cases and normal control patients. ***p<0.001.

Furthermore, we tested whether the expression of LINC00461 was deregulated in OA and whether LINC00461 expression was decreased in OA cases and normal control patients. We demonstrated that LINC00461 expression was higher in OA tissues than in normal samples (Figure 2, ***p<0.001).

### The expression of miR-30a-5p was downregulated in OA tissues

**Figure 3:**
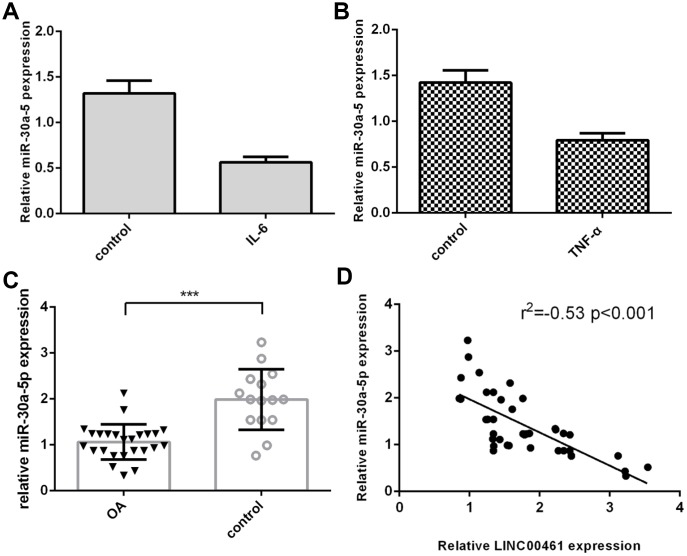
**The expression of miR-30a-5p was downregulated in OA tissues.** (**A**) IL-6 inhibited the expression of miR-30a-5p in chondrocytes. (**B**) TNF-α suppressed miR-30a-5p expression in chondrocytes. (**C**) The expression of miR-30a-5p was lower in OA tissues than in normal samples. (**D**) The expression level of miR-30a-5p in OA tissues was negatively correlated with LINC00461 expression. ***p<0.001.

We then demonstrated that IL-6 inhibited the expression of miR-30a-5p in chondrocytes (Figure 3A). We showed that TNF-α suppressed miR-30a-5p expression in chondrocytes (Figure 3B). In addition, we found that miR-30a-5p expression was lower in OA tissues than in normal samples (Figure 3C, ***p<0.001). The expression level of miR-30a-5p in OA tissues was negatively correlated with LINC00461 expression (Figure 3D).

### LINC00461 directly interacted with miR-30a-5p in chondrocytes

**Figure 4:**
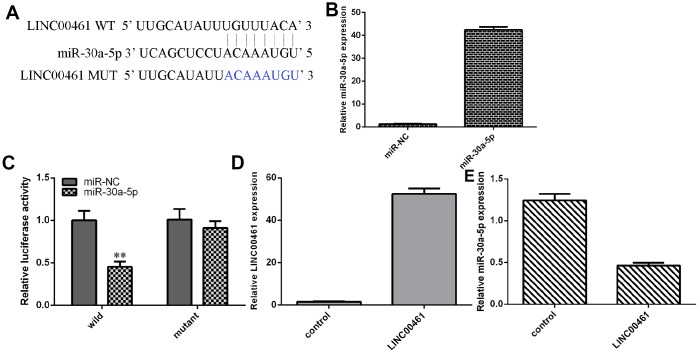
**LINC00461 directly interacted with miR-30a-5p in chondrocytes.** (**A**) There are complementary binding sites in the 3′-UTR (untranslated region) between LINC00461 and miR-30a-5p. (**B**) The expression of miR-30a-5p was upregulated in chondrocytes treated with miR-30a-5p mimics. (**C**) Overexpression of miR-30a-5p decreased the luciferase activity of WT-LINC00461 (wild type) but not Mut-LINC00461. (**D**) The expression of LINC00461 was analyzed by using qRT-PCR assay. (**E**) Elevated expression of LINC00461 suppressed the expression of miR-30a-5p in chondrocytes. **p<0.01.

As shown in Figure 4A, complementary binding sites were found in the 3′-UTR (untranslated region) between LINC00461 and miR-30a-5p by using starBase (http://starbase.sysu.edu.cn/index.php). We confirmed that the expression of miR-30a-5p was upregulated in chondrocytes treated with miR-30a-5p mimics (Figure 4B). In addition, we showed that overexpression of miR-30a-5p decreased the luciferase activity of WT-LINC00461 (wild type) but not Mut-LINC00461 (Figure 4C, **p<0.01). We also found that the expression of LINC00461 was upregulated in chondrocytes treated with pcDNA-LINC00461 (Figure 4D). Elevated expression of LINC00461 suppressed the expression of miR-30a-5p in chondrocytes (Figure 4E).

### Elevated expression of LINC00461 induced chondrocyte proliferation, cell cycle progression, inflammation, and extracellular matrix (ECM) degradation

**Figure 5:**
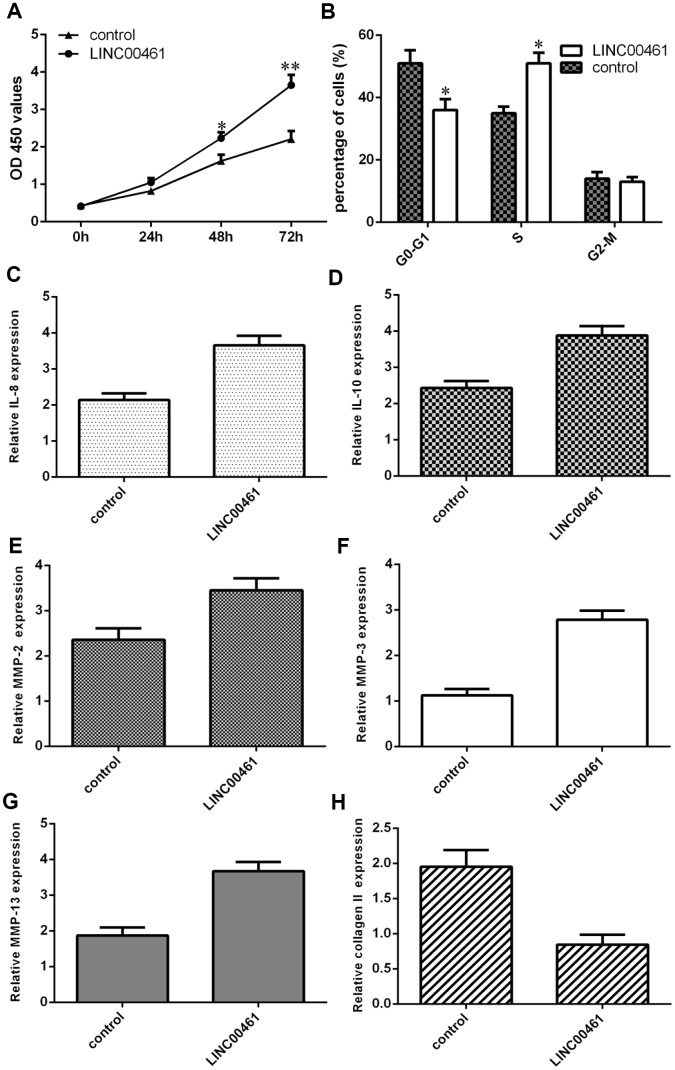
**Elevated expression of LINC00461 induced chondrocyte proliferation, cell cycle progression, inflammation, and extracellular matrix (ECM) degradation.** (**A**) Overexpression of LINC00461 enhanced cell proliferation in chondrocytes. (**B**) Elevated expression of LINC00461 increased cell cycle progression in chondrocytes. (**C**) IL-6 expression was upregulated in chondrocytes after LINC00461 treatment. (**D**) LINC00461 overexpression enhanced IL-10 expression in chondrocytes. (**E**) Ectopic expression of LINC00461 promoted MMP-2 expression in chondrocytes. (**F**) The expression level of MMP-3 was increased in chondrocytes after LINC00461 treatment. (**G**) Elevated expression of LINC00461 enhanced MMP-13 expression. (**H**) The expression of type II collagen was measured by qRT-PCR assay. *p<0.05 and **p<0.01.

Next, we showed that overexpression of LINC00461 significantly enhanced cell proliferation in chondrocytes (Figure 5A). We also demonstrated that elevated expression of LINC00461 increased cell cycle progression in chondrocytes (Figure 5B). Interestingly, we found that IL-6 expression was upregulated in chondrocytes after LINC00461 treatment (Figure 5C). Moreover, we showed that LINC00461 overexpression enhanced IL-10 expression in chondrocytes (Figure 5D). Ectopic expression of LINC00461 promoted MMP-2 expression in chondrocytes (Figure 5E). The expression level of MMP-3 was increased in chondrocytes after LINC00461 treatment (Figure 5F). Furthermore, we showed that elevated expression of LINC00461 enhanced MMP-13 expression (Figure 5G) and decreased type II collagen expression (Figure 5H) in chondrocytes.

### Ectopic expression of miR-30a-5p suppressed cell growth, cell cycle progression, inflammation and ECM degradation

**Figure 6:**
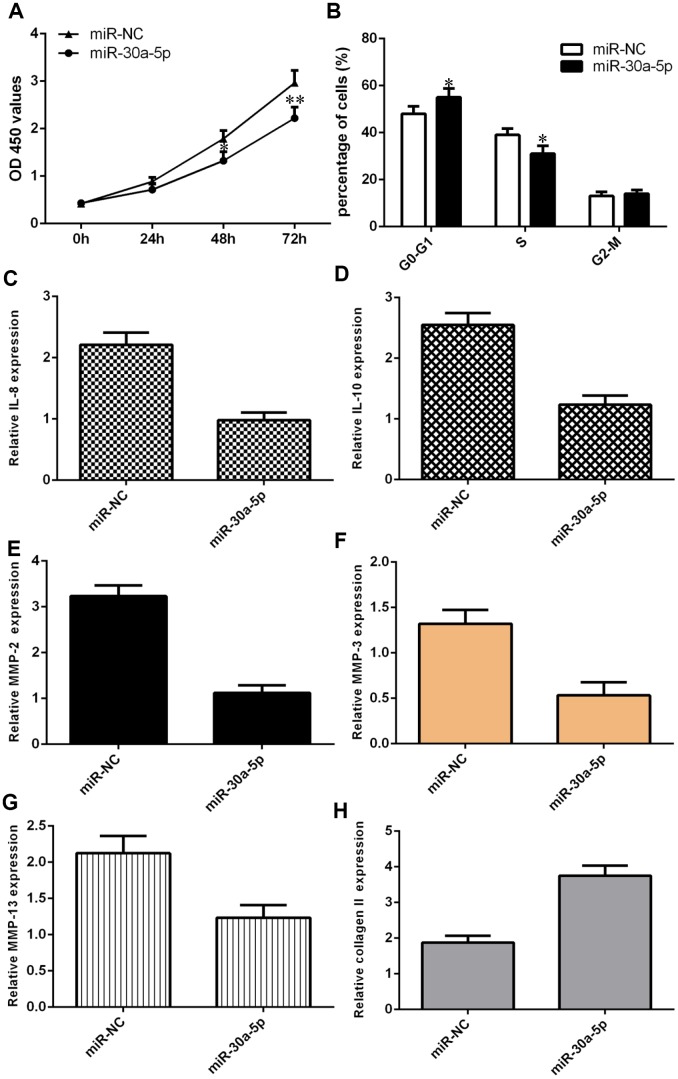
**Ectopic expression of miR-30a-5p suppressed cell growth, cell cycle progression, inflammation and ECM degradation.** (**A**) Overexpression of miR-30a-5p decreased cell proliferation in chondrocytes. (**B**) Elevated expression of miR-30a-5p suppressed the cell cycle in chondrocytes. (**C**) IL-6 expression was downregulated in chondrocytes after miR-30a-5p treatment. (**D**) miR-30a-5p overexpression inhibited IL-10 expression in chondrocytes. (**E**) Ectopic expression of miR-30a-5p decreased MMP-2 expression in chondrocytes. (**F**) The expression level of MMP-3 was decreased in chondrocytes after miR-30a-5p treatment. (**G**) Elevated expression of miR-30a-5p suppressed MMP-13 expression. (**H**) The expression of type II collagen was measured by qRT-PCR assay. *p<0.05 and **p<0.01.

We showed that overexpression of miR-30a-5p significantly decreased cell proliferation in chondrocytes (Figure 6A). We also demonstrated that elevated expression of miR-30a-5p suppressed the cell cycle in chondrocytes (Figure 6B). Interestingly, we found that IL-6 expression was downregulated in chondrocytes after miR-30a-5p treatment (Figure 6C). Moreover, we showed that miR-30a-5p overexpression inhibited IL-10 expression in chondrocytes (Figure 6D). Ectopic expression of miR-30a-5p decreased MMP-2 expression in chondrocytes (Figure 6E). The expression level of MMP-3 was decreased in chondrocytes after miR-30a-5p treatment (Figure 6F). Furthermore, we showed that elevated expression of miR-30a-5p suppressed MMP-13 expression (Figure 6G) and enhanced type II collagen expression (Figure 6H) in chondrocytes.

### Overexpression of LINC00461 enhanced chondrocyte proliferation, cell cycle progression, inflammation, and ECM degradation by downregulating miR-30a-5p

**Figure 7:**
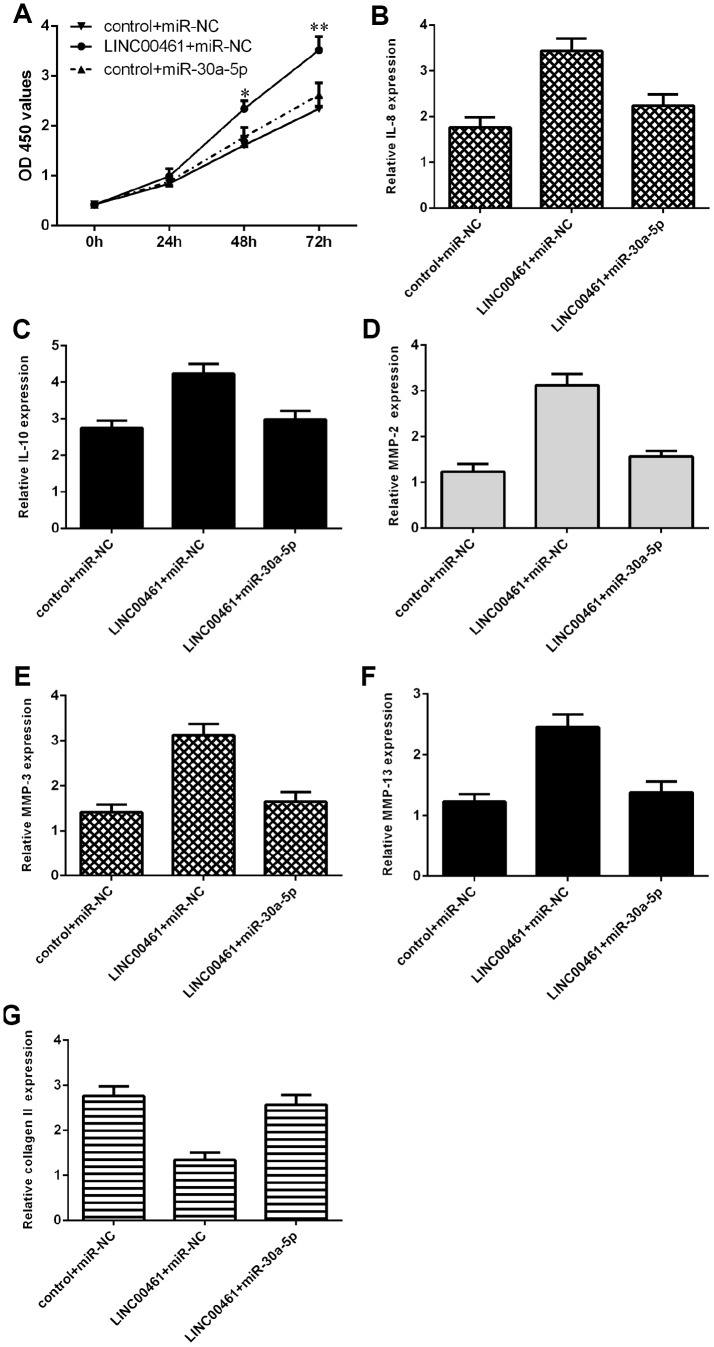
**Overexpression of LINC00461 enhanced chondrocyte proliferation, cell cycle progression, inflammation, and ECM degradation by downregulating miR-30a-5p.** (**A**) Cell proliferation was measured by CCK-8 assay. (**B**) Elevated expression of miR-30a-5p inhibited IL-8 in LINC00461-overexpressing chondrocytes. (**C**) The expression of IL-10 was determined by qRT-PCR assay. (**D**) Ectopic expression of miR-30a-5p decreased MMP-2 expression in LINC00461-overexpressing chondrocytes. (**E**) The expression of MMP-3 was measured by using qRT-PCR assay. (**F**) The expression of MMP-13 was measured by using qRT-PCR assay. (**G**) miR-30a-5p overexpression enhanced collagen II expression in LINC00461-overexpressing chondrocytes. *p<0.05 and **p<0.01.

We further explored whether LINC00461 promoted cells growth, inflammation, and ECM degradation by regulating miR-30a-5p in chondrocytes. We demonstrated that LINC00461 overexpression induced chondrocyte proliferation, while ectopic expression of miR-30a-5p significantly reversed this effect (Figure 7A). In addition, we showed that elevated expression of miR-30a-5p inhibited IL-8 (Figure 7B) and IL-10 (Figure 7C) expression in LINC00461-overexpressing chondrocytes. Furthermore, we showed that ectopic expression of miR-30a-5p suppressed MMP-2 (Figure 7D), MMP-3 (Figure 7E) and MMP-13 (Figure 7F) expression in LINC00461-overexpressing chondrocytes. We demonstrated that miR-30a-5p overexpression enhanced collagen II expression in LINC00461-overexpressing chondrocytes (Figure 7G).

## DISCUSSION

Emerging reports have demonstrated that lncRNAs are involved in the development of OA [2, 35, 36]. In our study, we demonstrated that the inflammatory mediators IL-6 and TNF-α induced LINC00461 expression in chondrocytes and that the expression of LINC00461 was upregulated in OA tissues. Furthermore, we showed that TNF-α and IL-6 suppressed the expression of miR-30a-5p and that miR-30a-5p expression was lower in OA tissues than in normal samples. The expression level of miR-30a-5p in OA tissues was negatively correlated with LINC00461 expression. In addition, we showed that LINC00461 directly interacted with miR-30a-5p in chondrocytes. Elevated expression of LINC00461 induced chondrocyte proliferation, cell cycle progression, inflammation, and extracellular matrix (ECM) degradation. However, we demonstrated that ectopic expression of miR-30a-5p suppressed cell growth, cell cycle progression, inflammation and ECM degradation. Finally, we found that overexpression of LINC00461 enhanced chondrocyte proliferation, cell cycle progression, inflammation, and ECM degradation by downregulating miR-30a-5p. These data demonstrated that LINC00461 may modulate the development of OA by suppressing miR-30a-5p expression in chondrocytes.

Accumulating studies have revealed that LINC00461 plays important roles in the progression of several tumors, including glioma, multiple myeloma and breast cancer [32, 33]. For instance, Yang et al. [32]. found that LINC00461 was overexpressed in glioma samples and that knockdown of LINC00461 inhibited the expression of cyclin D1/A/E and cell growth in glioma cells partly by regulating the PI3K/AKT and MAPK/ERK signaling pathways. In addition, LINC00461 knockdown suppressed the expression of miR-9 and the related genes TMEM161B and MEF2C. Deng et al. [33]. indicated that LINC00461 expression was upregulated in multiple myeloma and that LINC00461 knockdown reduced multiple myeloma cell proliferation and promoted cell apoptosis by regulating miR-15a and miR-16. Dong et al. [34]. demonstrated that LINC00461 was overexpressed in breast cancer cell lines and samples and that LINC00461 overexpression induced breast cancer cell invasion and migration, enhanced ZEB1 and vimentin expression and inhibited E-cadherin expression. However, the functional role of LINC00461 in OA development remains unknown. In our research, we first confirmed that the inflammatory mediators IL-6 and TNF-α induced LINC00461 expression in chondrocytes and that the expression of LINC00461 was upregulated in OA tissues. Elevated expression of LINC00461 induced chondrocyte proliferation, cell cycle progression, inflammation, and extracellular matrix (ECM) degradation. These data suggested that LINC00461 induced the progression of OA.

Previous studies have suggested that miRNAs are modulated by lncRNAs and play critical roles in the functional activity of lncRNAs [37–39]. For example, LINC00461 plays an oncogenic role in breast cancer by regulating miR-30a-5p expression [34]. Liu et al. found that lncRNA THRIL enhanced lipopolysaccharide-induced inflammatory injury by inhibiting miR-125b in ATDC5 cells. Xiao et al. [40]. showed that lncRNA HOTAIRM1 variant 1 downregulation contributes to OA by modulating miR-125b/BMPR2 expression and promoting the JNK/MAPK/ERK signaling pathway. Hu et al. [41]. showed that lncRNA HOTAIR induced OA development by regulating miR-17-5p and FUT2/β-catenin axis expression. In line with these findings, we also demonstrated that LINC00461 directly interacted with miR-30a-5p in chondrocytes. Moreover, we showed that TNF-α and IL-6 suppressed the expression of miR-30a-5p and that miR-30a-5p expression was lower in OA tissues than in normal samples. The expression level of miR-30a-5p in OA tissues was negatively correlated with LINC00461 expression. Ectopic expression of miR-30a-5p decreased cell growth, cell cycle progression, inflammation and ECM degradation. Furthermore, we found that overexpression of LINC00461 enhanced chondrocyte proliferation, cell cycle progression, inflammation, and ECM degradation by downregulating miR-30a-5p. We will study the mechanism/pathway of how LINC00461 exerts its potential effects in OA chondrocytes in our next work.

In summary, we demonstrated that LINC00461 was overexpressed in OA tissues compared to normal samples and that IL-6 and TNF-α induced LINC00461 expression in chondrocytes. Overexpression of LINC00461 enhanced chondrocyte proliferation, cell cycle progression, inflammation, and ECM degradation by downregulating miR-30a-5p. These findings suggested that lncRNA LINC00461 promoted the progression of OA partly by regulating miR-30a-5p expression.

## MATERIALS AND METHODS

### Tissues

OA cartilage samples were obtained from OA cases (n=25, age 61.04 ± 4.809 years; 18 female, 7 male) that underwent total knee arthroplasty, and control cartilage samples (n=15, age 43.5±4.2 years; 9 female, 6 male) were collected from cases that underwent amputation without rheumatoid arthritis or OA history (Supplementary Table 1). This research was approved by the Clinical Ethics Committee of the Affiliated Hospital of Jining Medical University, and all participants signed informed consent forms.

### Cell culture and treatment

Chondrocytes were separated from cartilage tissues of OA patients according to a previous protocol [42]. In brief, cartilage tissues were dissected into small sections and digested with trypsin (0.25%). Then, these tissues were incubated with type II collagenase (0.2%) and cultured in DMEM (Dulbecco’s modified Eagle’s medium) containing FBS. MiR-30a-5p mimic, miR-NC, pcDNA-LINC00461 and the pcDNA control were obtained from GenePharma (Shanghai, China) and transfected into cells by using Lipofectamine 2000 (Invitrogen, MA) at a concentration of 10 nmol/l following the manufacturer’s instructions.

### Quantitative real-time PCR (qRT-PCR)

Total RNA from cells or samples was separated with a TRIzol kit (Invitrogen, USA) and then reverse transcribed. qRT-PCR assay was performed to detect the expression of LINC00461, miR-30a-5p, IL-6, IL-10, MMP-2, MMP-13 and type II collagen using SYBR Green Mix (Qiagen, Germany) on the PCR system Biosystems 7300 (Foster City, USA). To create the melting curve, these reactions were set up at 95°C for 30 seconds followed by 46 cycles of 95°C for 5 seconds, 60°C for 10 seconds, and 72°C for 30 seconds. GAPDH and U6 were used as endogenous controls. Relative quantification of these genes was analyzed by the 2^-ΔΔCT^ method. The primer sequences were as follows: LINC00461, sense 5′-TTTTCCCCATAGTAATCG-3′; antisense 5′-ACTGGTTGATGTCGTTTG-3′; GAPDH, sense 5′-GGGAGCCAAAAGGGTCATCA-3′; antisense 5′-TGATGGCATGGACTGTGGTC-3′; miR-30a-5p sense 5′-ACACTCCAGCTGGGTGTAAACATCCTCGAC-3′; antisense 5′-CAGTGCGTGTCGTGGAGT-3′; and U6 sense 5′-CGCGCTTCGGCAGCACATATACT-3′; antisense 5′-ACGCTTCACGAATTTGCGTGTC-3′.

### Cell proliferation

Cell Counting Kit-8 (CCK-8, Dojindo, Japan) was used to analyze cell proliferation. Cells (approximately 5 × 10^3^) were cultured in 96-well plates. Cell growth was determined at different time points (0, 24, 48 and 72 hours). The absorbance at 450 nm was measured on a microplate reader (Pforzheim, Germany).

### Luciferase reporter assay

The 3′-UTR luciferase reporter construct of LINC00461 was constructed with a mutant and wild type sequence. Chondrocytes were cultured in 24-well plates and transfected with miR-30a-5p mimics, miR-NC and mutant-LINC00461 or wild-type-LINC00461 by using Lipofectamine 2000 (Invitrogen, MA) following the manufacturer’s instructions. Reporter assays were performed using a dual luciferase assay (Promega). The firefly luciferase activity was normalized to Renilla luciferase activity, and the firefly/Renilla ratio was recorded.

### Statistical analysis

The data of these experiments are shown as the mean±SD (standard deviation). SPSS software (version 19.0, IBM, NY, USA) was used to determine the statistical analysis. Differences between two groups were analyzed by Student's t-test. The correlation between the expression of miR-30a-5p and LINC00461 was analyzed by Pearson’s test. P < 0.05 was set as statistically significant.

## Supplementary Material

Supplementary Table 1
