# Supplementary material for: Long noncoding RNA LINC00461 induced osteoarthritis progression by inhibiting miR-30a-5p
Source: Aging (Albany NY). 2020 Mar 10;12(5):4111–23. doi: 10.18632/aging.102839 (PMC7093191; doi:10.18632/aging.102839)
Supplement: Supplementary Table 1 [file aging-12-102839-s001..pdf]

SUPPLEMENTARY TABLE

Supplementary Table 1. Clinical features of OA patients.

Clinicopathological features	Number
Age (years)	
<60	10
≥60	15
Gender	
Male	7
Female	18
Disease duration in months	
<60	8
≥60	19
Kellgren-Lawrence stage	
III	13
IV	12
